# Does Adding LEAP to ACL Reconstruction Reduce Graft Failure? A Systematic Review and Meta-Analysis of Comparative Studies with Minimum Two-Year Follow-Up

**DOI:** 10.3390/jcm14238499

**Published:** 2025-11-30

**Authors:** Simone Giusti, Angelo Matteucci, Simone Pavone, Ciro Mignano, Ezio Adriani

**Affiliations:** 1 UOC Traumatologia Dello Sport e Chirurgia Articolare, Università Cattolica del Sacro Cuore, Largo Francesco Vito 1, 00168 Rome, Italy; simonegiustiwork@gmail.com (S.G.); ciromignanotri@gmail.com (C.M.);; 2 IRCCS Humanitas Research Hospital, Via Manzoni 56, Rozzano, 20089 Milan, Italy; 3 Department of Biomedical Sciences, Humanitas University, Via Rita Levi Montalcini 4, Pieve Emanuele, 20072 Milan, Italy

**Keywords:** ACL reconstruction, sports medicine, lateral extra-articular procedure, LEAP, graft failure, knee stability, meta-analysis

## Abstract

**Background:** Anterior cruciate ligament (ACL) reconstruction (ACLR) is the gold-standard treatment for ACL rupture; however, graft failure remains a significant concern, particularly in young, active, or high-risk patients. The addition of a Lateral Extra-Articular Procedure (LEAP) has been proposed to enhance rotational stability and reduce the risk of graft rupture. This systematic review and meta-analysis aimed to determine whether adding LEAP to primary ACLR reduces graft failure rates compared to isolated ACLR, with a minimum follow-up of two years. **Methods:** A comprehensive literature search was conducted across PubMed, Scopus, Embase and Cochrane Library databases in accordance with PRISMA guidelines. Eighteen comparative studies with a minimum 24-month follow-up were included, encompassing 7336 patients (3857 undergoing isolated ACLR and 3479 undergoing ACLR with LEAP). The primary outcome was graft failure rate. Data were analyzed using the Mantel–Haenszel method with a fixed-effects model to calculate pooled risk ratios (RR) and 95% confidence intervals (CI). **Results:** Pooled analysis demonstrated a significant increase in graft rupture risk in the ACLR-alone group compared to ACLR + LEAP (RR 2.72; 95% CI: 2.23–3.32; *p* < 0.00001), with negligible heterogeneity (I^2^ = 0%). LEAP was particularly beneficial in high-risk populations such as young athletes and individuals with high-grade pivot shift or ligamentous laxity. Secondary outcomes showed improved rotational stability and comparable patient-reported outcomes and complication rates between groups. **Conclusions:** ACLR alone presents a higher risk of graft failure in comparison with the ALCR + LEAP technique. Furthermore, the addition of LEAP to ACLR improves rotational stability without increasing complications. LEAP should be selectively considered in patients with high-risk profiles or persistent intraoperative instability. These findings support the incorporation of LEAP as a valuable adjunct in modern ACL reconstruction strategies.

## 1. Introduction

Anterior cruciate ligament (ACL) tears are among the most common knee injuries, affecting more than 150,000 people each year, most of whom are athletes. Anterior cruciate ligament (ACL) reconstruction has become the gold standard for the treatment of ACL injuries. Over the years, various reconstruction techniques have been proposed and gradually improved. Among the various surgical techniques proposed, lateral extra-articular procedures (LEAP) are often used to address residual femoral rotation and the potential knee instability that may persist following anterior cruciate ligament (ACL) reconstruction [1]

Several recent studies have investigated the benefits of combining LEAP with ACLR, suggesting potential advantages in selected patient populations [2]. Currently, LEAP is typically indicated in the majority of cases involving high-grade rotational instability, anterolateral ligament injury, high-risk patients such as young athletes that practice pivoting sports (soccer, basketball, rugby) or those with ligamentous laxity, revision ACL reconstructions, and persistent rotational instability despite adequate intra-articular repair, although it is not routinely performed by all surgeons [1].

The aim of our systematic review and meta-analysis is to assess whether adding LEAP to primary ACL reconstruction significantly reduces graft failure rates compared to isolated ACLR, at a minimum follow-up of two years. Our analysis focuses on providing evidence-based guidance on the necessity and appropriateness of LEAP, considering that its routine application may not confer additional protective benefits and could represent an unnecessary surgical intervention in many cases.

## 2. Methods and Materials

This systematic review and meta-analysis adhered to the guidelines outlined in the Preferred Reporting Items for Systematic Reviews and Meta-analyses (PRISMA) The PRISMA checklist is provided in Supplementary Materials. The protocol for this systematic review and meta-analysis was prospectively registered in PROSPERO (ID: CRD420251158621).

### 2.1. Eligibility Criteria

(1) Cohort studies, Randomized clinical trials, Systematic reviews, Randomized comparative studies, Meta-analyses, Randomized controlled trials, Retrospective comparative studies, Retrospective propensity-matched cohort study, Retrospective cohort studies, Prospective randomized controlled trials, Retrospective cohort; propensity-matched case–control studies;
(2) Studies written in the English language;
(3) Studies on patients who underwent ACL reconstruction, with or without Lateral Extra-articular Procedures (LEAP);
(4) Only studies with patients who underwent ACL reconstruction with a minimum 24 months follow-up were considered;
(5) Only studies presenting graft rupture rate, in percentage (%) or in absolute value;

### 2.2. Exclusion Criteria

(1) Case reports, reviews, letters to the editor, cadaveric studies, commentaries, and editorials;
(2) All studies with Level of Evidence = 4 or 5;
(3) Mean age < 14 years;
(4) Studies older than 8 years (1 May 2018–1 May 2025);
(5) Revision reconstruction;

Data on type of graft used (Hamstring Tendons (HT), Bone-Patellar-Tendon Bone (BTB), Quadriceps Tendon (QT)), type of femoral tunnel practised (trans-tibial, modified trans-tibial or antero-medial tunnel) and type of bundle (single or double bundle) were not considered as exclusion criteria.

### 2.3. Information Sources and Search Strategy

A comprehensive search of PubMed, Scopus, Embase and the Cochrane Library was performed. Two reviewers (M.A. and P.S.) independently gathered studies, and then the results were cross-checked and merged for analysis.

All search results were independently screened by title/abstract and then by full text against predefined criteria (clinical studies reporting graft rupture rate after ACL reconstruction, in percentage or in absolute values).

A total of 214 records were identified through database searching. After removal of 7 duplicates and screening of titles and abstracts, 177 studies were excluded. 37 full-text articles were assessed for eligibility, of which 19 were excluded for reasons such as non-comparative design, follow-up shorter than 2 years, or pediatric cohorts. 18 studies met the inclusion criteria and were included in the final qualitative and quantitative synthesis.

### 2.4. Data Items

Extracted data consisted of the following:

(1) Study information: author, publication year, title, study type, level of evidence, purpose, and main conclusions;
(2) Population details: sample size, sex distribution (when possible), mean age, and therapeutic protocol (ACL reconstruction, with or without LEAP);
(3) Methodology specifics: type of graft (when possible), graft rupture rate (directly in percentage % or indirectly, as event occurred/number of participants);

### 2.5. Risk of Bias

P.S. and M.C. performed the risk-of-bias assessment independently and in duplicate, using tools appropriate to study design: RoBANS for non-randomized studies and RoB 2 for randomized trials. Any disagreements were resolved by discussion; if consensus was not reached, a third reviewer (M.A.) adjudicated. RoBANS comprises six domains—(a) patient selection, (b) confounding variables, (c) measurement of exposure, (d) blinding of outcome assessment, (e) incomplete outcome data, and (f) selective outcome reporting—each rated Low, High, or Unclear risk. RoB 2 includes five domains—(1) randomization process, (2) deviations from intended interventions (effect of assignment), (3) missing outcome data, (4) measurement of the outcome, and (5) selection of the reported result—with signaling questions supporting domain judgments (Low risk, Some concerns, High risk).

Overall judgment rules followed standard criteria: Low risk if all domains were Low; Some concerns if no domain was High but ≥1 domain raised concerns; High risk if ≥1 domain was High or multiple concerns substantially lowered confidence.

Risk of bias was assessed for the 18 included studies: five RCTs (assessed with RoB 2) and thirteen non-randomized studies (assessed with RoBANS). Among the RCTs, Heard et al. [3], Getgood et al. [4], and Porter et al. [5] were judged Low risk across all RoB 2 domains, while El-Azab et al. and Lucidi et al. raised some concerns in certain domains, resulting in an overall judgment of some concerns.

RoBANS assessments indicated heterogeneous risk-of-bias patterns among non-randomized studies. Patient selection, exposure ascertainment, completeness of outcome data, and selective reporting were generally judged at low risk. However, confounding and outcome assessment frequently raised concerns, primarily due to unmeasured confounders and the absence of blinding procedures, which could influence outcome evaluation. Notably, Monaco et al. [6], Viglietta et al. [7], Serna et al. [8], Mahmoud et al. [9], and Rowan et al. [10], were low across all RoBANS domains (overall low risk). Risk of bias due to missing results was assessed visually using funnel plot: suggested slight asymmetry, indicating a possible risk of publication bias.

**Figure 1:**
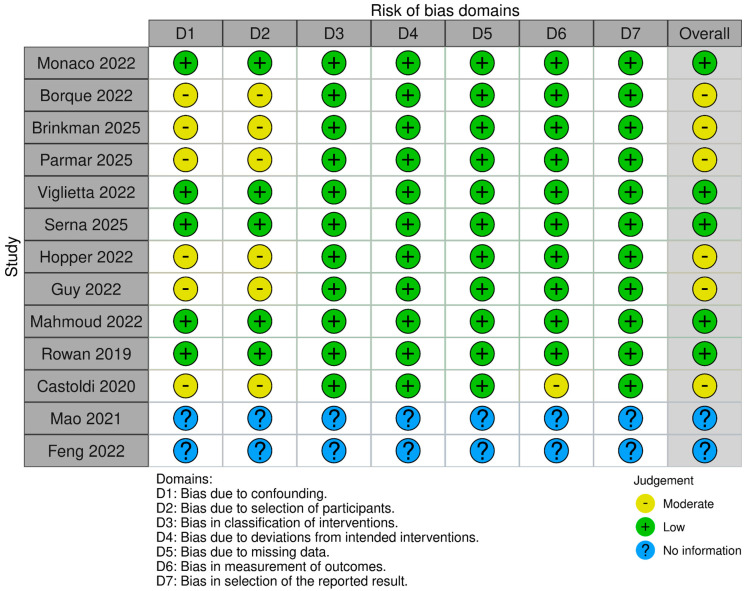
Risk of Bias—Non-Randomized Studies (RoBANS) by robvis [6,7,8,9,10,11,12,13,14,15,16,17,18].

**Figure 2:**
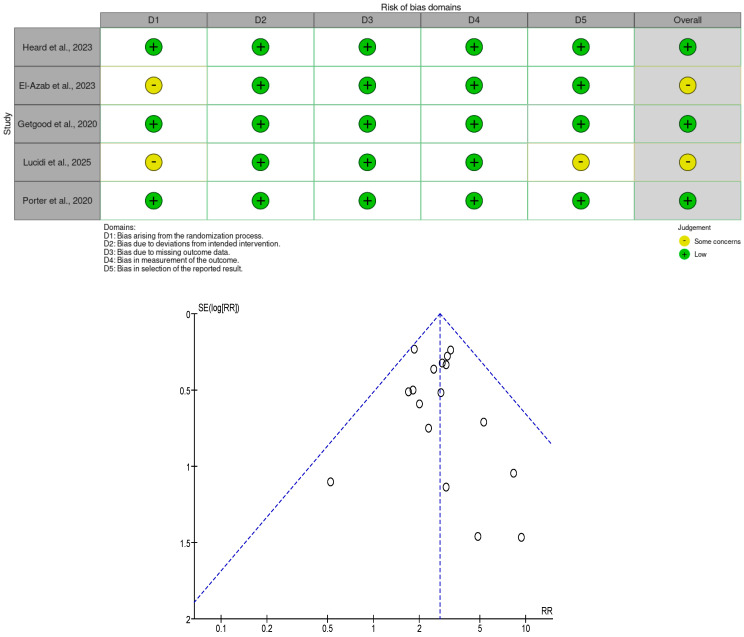
Risk of Bias—Randomized Controlled Trials (RoB 2) by robvis [3,4,5,19,20]. Risk of Publication Bias—Funnel plot.

Visual summaries are provided in Figure 1 and Figure 2.

### 2.6. Data Analysis

Patients from the included studies were categorized into two groups based on the type of technique practiced (LEAP or non-LEAP).

Successively, each single event was extracted from the rupture’s percentage. When they occurred, results were rounded to excess or defect. If a study presented more graft-harvesting techniques, they were summed, and considered as a single result.

Our aim was to calculate the risk ratio of each group, and then a comparison was made between the two sets of data.

All events were inserted in Review Manager software (RevMan, version 5.4.1, Cochrane, London, UK), using dichotomous data with Mantel-Haenzsel statistical method, risk ratio on fixed effect (I^2^ = 0%), IC 95%.

The degree of heterogeneity in graft rupture rate was assessed with the I^2^ index, which was interpreted as follows: 0.0–24.9% to indicate no heterogeneity, 25.0–49.9% to indicate low heterogeneity, 50.0–74.9% to indicate moderate heterogeneity, 75.0–100.0% to indicate high heterogeneity. A higher I^2^ score implies a larger proportion of variability in the results that could be attributed to heterogeneity [16].

Sensitivity analyses using a random-effects model produced similar pooled estimates (Risk Ratio 2.62, 95% CI 2.14–3.19; Tau^2^ = 0.00; Chi^2^ = 12.57, df = 17 (*p* = 0.76); I^2^ = 0%, *p* < 0.00001), confirming the robustness of the fixed-effects results.

In addition, the pooled risk difference (random-effects model) was 0.07 (95% CI 0.05–0.09), with moderate heterogeneity (I^2^ = 63%). This variability likely reflects differences in baseline event rates across studies rather than inconsistent treatment effects, as the relative effect (RR) was homogeneous (I^2^ = 0%).

To address differences in sample size across studies, weighted mean values were used instead of arithmetic mean values. The use of weighted mean values allowed studies with larger sample sizes to contribute more to the computed average compared to studies with small sample sizes.

Certainty of the evidence was not formally assessed using the GRADE approach; however, potential limitations, risk of bias, and inconsistency among included studies were qualitatively discussed to provide an overall appraisal of the confidence in the findings

### 2.7. Study Selection and PRISMA Flow

**Figure 3:**
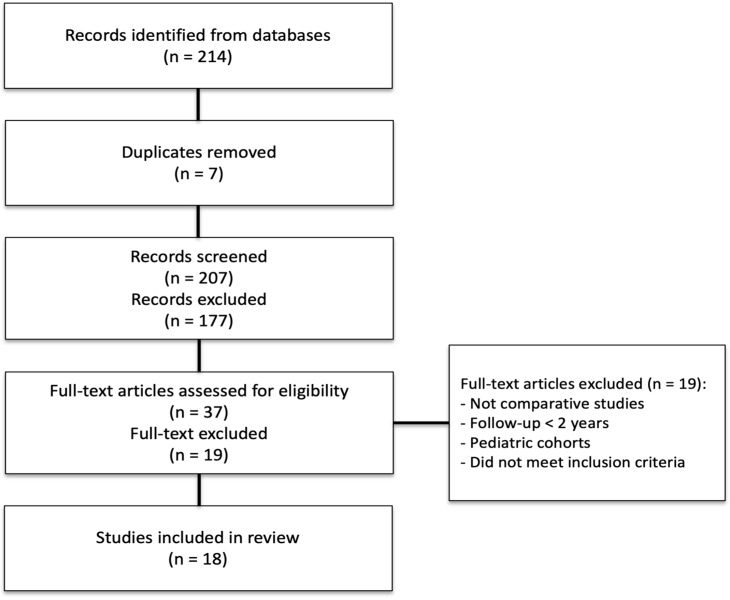
PRISMA 2020 flow diagram summarizing the study selection process. A total of 214 records were identified through database searching. After removal of 7 duplicates and screening of titles and abstracts, 177 studies were excluded. Thirty-seven full-text articles were assessed for eligibility, of which 19 were excluded for reasons such as non-comparative design, follow-up shorter than 2 years, or pediatric cohorts. Eighteen studies met the inclusion criteria and were included in the final qualitative and quantitative analyses.

The systematic review was conducted in accordance with the Preferred Reporting Items for Systematic Reviews and Meta-Analyses (PRISMA) 2020 guidelines. Study screening and selection were performed independently by two reviewers (S.P. and A.M.). Any disagreements regarding inclusion or exclusion were resolved through discussion. In cases where consensus could not be reached, a third reviewer (C.M.) was consulted, and their decision was considered final. The search strategy identified 214 records, of which 7 duplicates were removed. After screening titles and abstracts, 177 records were excluded. A total of 37 full-text articles were assessed for eligibility, and 19 were excluded for not meeting the predefined inclusion criteria (e.g., not comparative studies, follow-up <2 years, pediatric cohorts). Eighteen studies met all criteria and were included in the final analysis. The study selection process is detailed in the PRISMA flow diagram. (Figure 3).

## 3. Results

Eighteen comparative studies meeting inclusion criteria were analyzed, encompassing a total of 7336 patients (3857 undergoing isolated ACL reconstruction [ACLR] and 3479 undergoing ACLR with additional lateral extra-articular procedures [ACLR + LEAP]). All included studies reported graft rupture rates at a minimum of 2-year follow-up, with patient populations generally composed of high-risk individuals (e.g., young athletes, those with high-grade pivot shift or generalized ligamentous laxity).

Across individual studies, the incidence of graft failure varied markedly between groups. In 17 of the 18 studies, the addition of LEAP was associated with numerically lower re-rupture rates compared to ACLR alone. For instance, Monaco et al. [6] reported 0% graft failure in the ACLR + LEAP group versus 15% in the ACLR-only group, while Hopper et al. [14] observed a significant difference between 6.0% and 15.5%, respectively (*p* = 0.0105). In contrast, Parmar et al. [13], was the only study reporting a slightly higher failure rate in the LEAP group (4.7% vs. 3%), though the difference was not statistically significant. Several high-quality RCTs (e.g., Getgood et al. [4], Heard et al. [3], El-Azab et al. [19]) consistently demonstrated a relative risk reduction in graft failure with LEAP augmentation (Table 1).

**Figure 4:**
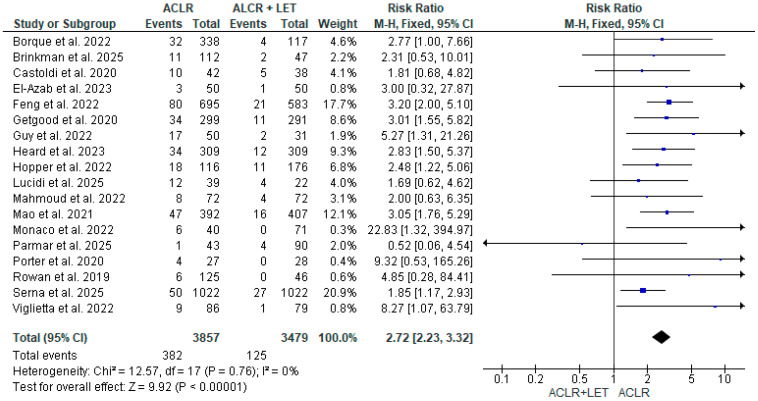
**Pooled analysis using a fixed-effect Mantel-Haenszel model** showed a significant increase in the risk of graft rupture in the ACLR alone group compared to ACLR + LEAP, with a risk ratio (RR) of 2.72 (95% CI: 2.23 to 3.32; *p* < 0.00001) [3,4,5,6,7,8,9,10,11,12,13,14,15,16,17,18,19,20].

Pooled analysis using a fixed-effect Mantel-Haenszel model showed a significant increase in the risk of graft rupture in the ACLR alone group compared to ACLR + LEAP, with a risk ratio (RR) of 2.72 (95% CI: 2.23 to 3.32; *p* < 0.00001). This suggests that patients undergoing isolated ACLR are nearly three times more likely to experience graft failure compared to those receiving an additional LEAP procedure. The heterogeneity among studies was negligible (I^2^ = 0%, Chi^2^ = 12.57, df = 17; *p* = 0.76), supporting the consistency of this finding across diverse populations and study designs (Figure 4).

Overall, the results of this meta-analysis confirm that the addition of LEAP to ACLR significantly reduces the risk of graft failure, particularly in high-risk populations. This benefit appears robust across study design, graft type, and surgical technique, and is achieved without a meaningful increase in adverse events. A summary of the results is included in Table 2.

## 4. Additional Findings

In addition to graft failure rates, secondary outcomes were consistently reported across the included studies and revealed converging trends favoring the addition of LEAP.

In terms of **rotational stability**, Monaco et al. [6] reported a pivot-shift grade 3 in 11.4% of the ACLR group versus 0% in the ACLR + LEAP group (*p* = 0.021), and a side-to-side anteroposterior laxity >5 mm in 17.1% versus 0% (*p* = 0.003). Similarly, Brinkman et al. [12], found reduced postoperative pivot-shift grades in both the HT + LEAP and QT groups compared to isolated HT (retear rate: 17.9% HT vs. 4.3% HT + LEAP). Viglietta et al. [7] also showed a markedly lower instability rate in the combined ACLR + LEAP group (graft failure: 1.2% vs. 10.4%).

Regarding **patient-reported outcomes**, Getgood et al. [4] and El-Azab et al. [19] observed no statistically significant differences in IKDC or KOOS scores between groups at final follow-up, while Porter et al. [5] documented superior Lysholm scores (96.8 vs. 92.5; *p* = 0.004) and Tegner activity scale (median 8 vs. 7; *p* = 0.03) in the LEAP group. Rowan et al. [10] found that patients receiving LEAP achieved a significantly higher median Lysholm score (98 vs. 90; *p* = 0.005) and returned to sport sooner (6 vs. 8 months; *p* < 0.001).

**Return to sport rates** were comparable or improved with LEAP augmentation. For instance, Heard et al. [3] and Hopper et al. [14], showed equivalent or better return-to-play metrics in the ACLR + LEAP group. In Rowan et al. [10], 100% of LEAP patients resumed pivoting sports versus 76% in the ACLR group.

**Adverse event rates** were similar between groups. Heard et al. [3] reported no significant increase in minor surgical or medical complications with LEAP. Mahmoud et al. [9] observed comparable complication profiles (e.g., 3 vs. 4 re-arthroscopies; 0 vs. 1 DVT), and no study reported increased incidence of postoperative stiffness or infection attributable to LEAP.

Finally, while one study (Castoldi et al. [16]) suggested a trend toward increased lateral compartment osteoarthritis after LEAP, no consistent association was found across the remaining long-term follow-up studies. On the contrary, Viglietta et al. [7] reported lower Kellgren–Lawrence OA grades in the LEAP group.

### Subgroup Analyses: Risk Populations and Graft Types

Subgroup analyses revealed notable patterns based on patient risk profiles and graft type.

**High-risk populations**, such as adolescents, elite athletes, and individuals with generalized ligamentous laxity or high-grade pivot shift, consistently benefitted from the addition of LEAP. For instance, in the STABILITY trial (Getgood et al. [4]), which enrolled patients ≤25 years old with ≥2 high-risk criteria, the 2-year graft rupture rate was significantly reduced in the LEAP group (3.7% vs. 11.3%; *p* < 0.01). Hopper et al. [14] demonstrated a hazard ratio (HR) of 2.678 (95% CI 1.173–4.837; *p* = 0.0164) for graft failure in isolated ACLR, with even stronger effects observed in athletes under 21 years (HR = 2.381; *p* = 0.0068). Rowan et al. [10], focusing on elite athletes, reported 0% re-ruptures in the LEAP group versus 5% in ACLR alone, and Guy et al. [15] showed a failure rate of 6.5% with LEAP versus 34% without in elite skiers (*p* = 0.0412).

In contrast, studies in lower-risk populations or those not explicitly stratified by risk (e.g., Parmar et al. [13], Mahmoud et al. [9]) did not show as pronounced a benefit, and in Parmar’s cohort of female soccer players, failure rates were slightly higher in the LEAP group (4.7% vs. 3%), though not significantly.

With regard to **graft type**, several studies stratified outcomes by the source of the autograft. Castoldi et al. [16] used BTB grafts and found a 29% failure rate in ACLR versus 13% in ACLR + LEAP. In contrast, Lucidi et al. [20] compared three techniques: BTB (37% re-rupture), hamstring tendon (25%), and over-the-top HT + LEAP (19%), indicating a potential protective effect of LEAP independent of graft type. Brinkman et al. [12] compared hamstring autografts (HT) and quadriceps tendon autografts (QT), observing a notably higher failure rate in HT (17.9%) compared to QT (1.8%) and showing that combining HA with LEAP (4.3%) substantially reduced this risk. Getgood et al. [4] and Heard et al. [3], both using hamstring autografts, reported improved stability and reduced re-rupture when augmented with LEAP, suggesting a particular benefit in this graft category.

**Table 2:** Summary of the results.

Publication	Study Design and Level of Evidence	Therapeutic Protocol	Outcomes	Patients’ Characteristics	Graft Rupture Rates	Additional Findings	Contra-Lateral YES/NO (Y/N)
Monaco et al., 2022 [6]	Cohort study; Level of evidence, 3	Early adolescent patients who underwent ACLR using a hamstring tendon autograft with (71) or without (40) the Arnold-Coker modification of the MacIntosh procedure; patients with ≥1 additional risk factors for a graft rupture were offered LEAP in addition to ACLR	Graft rupture rates, patient-reported outcome measure scores (KOOS and subjective IKDC), knee stability, return-to-sports rates, reoperation rates, and complications were assessed.	Patients (*n*): 111 (71 + 40) Age (Y): 16.3 (mean) Follow up: 24–89 months	ACLR: 15% ACLR + LEAP: 0% OR: 15.91 [95% CI, 1.81–139.44]; *p* = 0.012)	Significantly better knee stability with ACLR + LEAP (pivot-shift grade 3: 0.0% vs. 11.4%, respectively; *p* = 0.021) (side-to-side anteroposterior laxity difference >5 mm: 0.0% vs. 17.1%, respectively; *p* = 0.003) and Tegner activity scores (7 vs. 6, respectively; *p* = 0.010).	N/D (not determined)
Borque et al., 2022 [11]	Cohort study; Level of evidence, 3	A consecutive cohort of elite athletes with an isolated ACL tear undergoing autograft patellar or hamstring tendon reconstruction with (117) or without (338) Lemaire LEAP	Graft rupture rate	Patients (*n*): 455 (117 + 338) Age (Y): 22.5 (mean) Follow up: 24 months	ACLR: 9.5% ACLR + LEAP: 3.4%	The addition of LEAP reduced the risk of undergoing revision by 2.8 times in elite athletes undergoing primary ACLR. This risk reduction did not differ significantly between the patellar tendon and hamstring tendon autografts. With these results, status as an elite athlete should be included in the indications for a LEAP, as they are at increased risk for ACL graft failure.	N/D
Heard et al., 2023 [3]	Randomized clinical trial; Level of evidence, 1	Stability is a randomized clinical trial comparing hamstring tendon ACLR with and without LEAP. Patients aged 14–25 years with an ACL-deficient knee were included. Patients were followed and adverse events documented (type, actions taken, resolution) with visits at 3, 6, 12, and 24 months postoperatively.	Adverse events were categorized as none, minor medical, minor surgical, contralateral ACL rupture, or graft rupture. Patient-reported outcome measures (PROMs) collected at each visit included the Knee Injury and Osteoarthritis Outcome Score (KOOS), International Knee Documentation Committee Score (IKDC), and ACL Quality of Life Questionnaire (ACL-QOL).	Patients (*n*): 618 (309 + 309) Age (Y): 18.9 (mean) Follow up: 24 months	ACLR: 11% ACLR + LEAP: 4%	The addition of LEAP to hamstring tendon autograft ACLR in young patients at high risk of re-injury resulted in a statistically significant reduction in graft rupture. While the addition of LEAP may increase rates of hardware irritation, there was no significant increase in overall rates of minor medical adverse events, minor surgical events, or overall re-operation rates.	N/D
Mao et al., 2021 [17]	Systematic review; Level of evidence, 2	PubMed, Embase, and the Cochrane Central Register of Controlled Trials databases were searched between inception and 1 July 2020. Level 1 or 2 randomized controlled trials that compared isolated single-bundle ACLR with (407) or without (392) LEAP with ACLR were included.	Data were meta-analyzed for the primary outcome measure of knee stability and the secondary outcome measures of patient-reported outcome scores, return to sports, and graft failure. Dichotomous variables were presented as relative risks (RRs), and continuous variables were presented as mean differences (MDs) and standardized MDs (SMDs).	Patients (*n*): 799 (392 + 407) Age (Y): 18.8–31.3 (mean) Follow up: 24 months min	ACLR: 12% ACLR + LEAP: 3.93%	The addition of LEAP to single-bundle ACLR appeared to be associated with a statistically significant, clinically relevant reduction in postoperative ALRI in the long term (>2 years) relative to ACLR alone. The adoption of LEAP may also lead to higher postoperative activity levels and a lower incidence of graft failure. For these reasons, the LEAP procedure should be considered in combination with isolated single-bundle ACLR, particularly in patients involved in strenuous sports.	N/D
El-Azab et al., 2023 [19]	Randomized Comparative Study; Level of evidence, 1	A Prospective Blinded Randomized Controlled study included 100 consecutive patients who underwent ACL-R with hamstring tendon grafts in our Hospital. The patients were allocated into two groups (Group A and B) with randomization; group A received ACL-R with a large-size ACL-graft diameter of 6 strands, and group B received ACL-R of 4 strands combined with lateral extraarticular tenodesis (LEAP) (Modified Lemaire). Each group had fifty patients.	They were examined for graft failure, anterolateral rotatory instability with the pivot shift test, and clinical outcomes, which were evaluated with the International Knee Documentation Committee score (IKDC) both subjective and objective.	Patients (*n*): 100 (50 + 50) Age (Y): 27.5 (mean) Follow up: 24 months	ACLR: 6.3% ACLR + LEAP: 2.1%	Both groups showed a low ACL-graft failure rate, low anterolateral rotatory instability, and a good clinical outcome. Although there was no significant difference in subjective IKDC score between both groups, the failure rate and anterolateral rotatory instability were significantly lower in the ACL-R (4 strands) with LEAP combination group than in the group with the large-diameter (6 strands) graft.	N/D
Feng et al., 2022 [18]	Systematic review and meta-analysis; Level of evidence, 1	PubMed, Embase, and Cochrane Library were searched by two researchers for clinical studies comparing ACLR with (583) and without LEAP (695). Studies with only evidence levels I and II and studies in which anterior lateral ligament reconstruction was performed with grafts were excluded. The risk of bias of the studies was assessed using the Cochrane risk-of-bias and modified Downs & Black tools.	The outcomes included (1) functional outcomes; (2) knee laxity measures; (3) knee injury osteoarthritis and outcome score; and (4) complications. The outcomes of the two groups were extracted, summarized and compared.	Patients (*n*): 1278 (583 + 695) Age (Y): 22.8 (mean) Follow up: 30 months	ACLR: 11.5% ACLR + LEAP: 3.6%	ACLR combined with LEAP can effectively reduce rotation laxity of the knee joint and reduce the graft failure rate in high-risk patients. However, the effects on the function and activity level of patients cannot be confirmed.	N/D
Castoldi et al., 2020 [16]	Randomized controlled trial; Level of evidence, 2	This study included 121 consecutive knees (120 patients) presenting to a single center with an ACL rupture between 1998 and 1999. In total, 61 knees were randomized to an isolated BTB ACLR, and 60 knees were randomized to a BTB ACLR with an extra-articular lateral tenodesis with gracilis tendon (modified Lemaire). Eighty knees in 79 patients (66%) were available for follow-up at a postoperative mean of 19.4 years (range, 19–20.2). Of those patients, 43 had a clinical examination and completed patient-reported outcome questionnaires, and the other 37 patients were evaluated through the questionnaires alone.	Standard radiographs were available for 45 patients and laximetry (TELOS) for 42 patients. Mean subjective International Knee Documentation Committee score at last follow-up was 81.8, and no differences were noted between the BTB and BTB-LEAP groups (*p* = 0.7). Two-thirds of patients were still participating in pivoting sports.	Patients (*n*): 80 (38 + 42) Age (Y): 26.2 mean Follow up: 19.4 years (mean)	ACLR: 29% ACLR + LEAP: 13%	There were no significant differences in long-term patient-reported outcomes after ACLR with or without an LEAP. LEAP may increase the risk of lateral compartment osteoarthritis at long-term follow-up. There was a trend toward decreased graft failure risk with the addition of LEAP but this study was underpowered to assess this outcome.	N/D
Getgood et al., 2020 [4]	Randomized controlled trial; Level of evidence, 1	This is a multicenter, prospective, randomized clinical trial comparing a single-bundle, hamstring tendon ACLR with (291) or without (299) LEAP performed using a strip of iliotibial band. Patients 25 years or younger with an ACL-deficient knee were included and also had to meet at least 2 of the following 3 criteria: (1) grade 2 pivot shift or greater, (2) a desire to return to high-risk/pivoting sports, (3) and generalized ligamentous laxity (GLL).	The primary outcome was ACLR clinical failure, a composite measure of rotatory laxity or a graft rupture. Secondary outcome measures included the P4 pain scale, Marx Activity Rating Scale, Knee injury Osteoarthritis and Outcome Score (KOOS), International Knee Documentation Committee score, and ACL Quality of Life Questionnaire. Patients were reviewed at 3, 6, 12, and 24 months postoperatively.	Patients (*n*): 590 (291 + 299) Age (Y): 18.9 years Follow up (m): 24 months	ACLR: 11.3% ACLR + LEAP: 3.7%	The number needed to treat with LEAP to prevent 1 patient from graft rupture was 14.3 over the first 2 postoperative years. At 3 months, patients in the ACLR group had less pain as measured by the P4 (*p* = 0.003) and KOOS (*p* = 0.007), with KOOS pain persisting in favor of the ACLR group to 6 months (*p* = 0.02). No clinically important differences in patient-reported outcome measures were found between groups at other time points. The level of sports activity was similar between groups at 2 years after surgery, as measured by the Marx Activity Rating Scale (*p* = 0.11).	N/D
Brinkman et al., 2025 [12]	Retrospective cohort study; Level of evidence, 3	A retrospective review was performed comparing high-risk patients undergoing ACL reconstruction with isolated HA (56), isolated QA (56), or HA + LEAP (47) from August 2013 to January 2023. High-risk patients, as determined by high-grade pivot shift or generalized ligament laxity, with at least a 2-year follow-up, were included.	Lysholm and International Knee Documentation Committee scores were compared at 3, 6, 12, and 24 months postoperatively. Retear rate, postoperative pivot-shift grade, return to sport, and complications were recorded.	Patients (*n*): 173 (56 + 56 + 47) Age (Y): 18.2 min (mean) Follow up: 24 months	ACLR: HA 17.9%, QA 1.8% ACLR + LEAP: 4.3%	The use of an all-soft tissue QA or HA + LEAP for ACL reconstruction resulted in a lower retear rate and postoperative pivot-shift grade compared to an isolated HA graft in high-risk patients at 2 years postoperatively. There was no difference in the rate of achieving the minimal clinically important difference between the cohorts. The QA and HA + LEAP reconstruction options may improve stability and decrease the failure rate compared with HA reconstruction alone.	N/D
Parmar et al., 2025 [13]	Retrospective comparative study; Level of evidence, 3	A retrospective review of female high school and collegiate soccer players who underwent primary ACLR from 2013 to 2021, with a minimum of 2 years of follow-up, was conducted. Participants were divided into 2 groups: those who received ACLR alone (43) and those who received ACLR with LEAP (90). Generalized ligamentous laxity was defined as a Beighton score ≥4 and was not considered an indication for LEAP. Anterior cruciate ligament (ACL) autografts included the quadriceps, bone-patellar tendon-bone, and hamstrings in both groups.	Patient demographics and physical examination findings, including pivot shift results, were collected. Positive pivot-shift refers to a grade ≥2. Patient outcomes included graft failure (defined as ACL retear), International Knee Documentation Committee score, Lysholm score, return to sport, and complications. Independent *t* tests, χ2 tests, and Mann-Whitney U tests were conducted to compare outcomes between the 2 groups. Minimally clinical important difference was calculated from preoperative to final follow-up.	Patients (*n*): 133 (43 + 90) Age (Y): 17.8 min (mean) Follow up: 24 months min	ACLR: 3% ACLR + LEAP: 4.7%	The addition of LEAP during ACLR in female soccer players with preoperative generalized ligamentous laxity yields graft retear and return-to-sport rates comparable with those of athletes without ligamentous laxity.	N/D
Lucidi et al., 2025 [20]	Randomized controlled trial; Level of evidence, 1	This study aimed to compare the failure rate, clinical outcomes, and OA incidence of 3 different ACL reconstruction techniques: single-bundle quadrupled hamstring tendon (HT) (20), bone-patellar tendon-bone (BPTB) (19), and over-the-top HT plus LEAP (HT + LEAP) (22). The authors hypothesized that the 3 techniques would have comparable clinical and radiographic outcomes at long-term follow-up.	At the last follow-up (minimum of 20 years), patient-reported outcome measure (PROM) scores, complications, and reoperations were collected, and an objective clinical evaluation was performed, including the measurement of anteroposterior (AP) laxity using an arthrometer and the quantification of the pivot shift (PS) using a triaxial accelerometer. Clinical failure was considered in patients with evidence of a graft rupture or those with a side-to-side difference in AP laxity >5 mm or with a side-to-side difference in the PS >1.5 mm/s^2^.	Patients (*n*): 61 (19, 20, 22) Age (Y): 29.3 (mean) Follow up: 22.9 years min	ACLR: 37% (BPTB) 25% (HT) ACLR + LEAP: 19%	The 3 investigated techniques (BPTB, quadrupled HT, and over-the-top HT plus LEAP) provided comparable good clinical and radiographic outcomes at a mean follow-up time of 23 years. The BPTB group showed a greater prevalence of patellofemoral OA than the HT + LEAP group, while no difference was reported for tibiofemoral OA. The BPTB group revealed a slightly lower Tegner score than the HT + LEAP group, while the HT group showed slightly higher AP laxity than the BPTB group.	N/D
Viglietta et al., 2022 [7]	Cohort study; Level of evidence, 3	The study included 165 consecutive patients treated at a single center by ACLR. A total of 86 patients underwent iACLR (iACLR group) and 79 received combined intra- and extra-articular reconstruction (ACLR + LEAP).	The International Knee Documentation Committee (IKDC), Lysholm, and Tegner activity scores were administered. Knee stability was tested through the Lachman test, the pivot-shift test, and the KT-1000 knee arthrometer test. A positive pivot-shift test (++/+++), laxity on the KT-1000, and referred giving-way episodes or revision ACLR were considered failures. Radiographic results were assessed according to the Fairbank, IKDC, and Kellgren-Lawrence scales. Radiographic evaluation included both the overall tibiofemoral joint and the medial and lateral compartments separately. A univariate and a multivariate logistic regression with penalized maximum likelihood was used to identify risk factors associated with long-term OA.	Patients (*n*): 165 (86 + 79) Age (Y): 26.1 (mean) Follow up: 15.7 years (mean)	ACLR: 10.4% ACLR + LEAP: 1.2%	A significantly higher risk of long-term OA was found with iACLR than with ACLR combined with the Arnold-Coker modification of the McIntosh extra-articular procedure. Knees with combined ACLR also had a significantly lower OA grade after partial lateral meniscectomy. Additionally, those undergoing combined ACLR had better knee stability and lower graft rupture rates at the long-term follow-up. Partial meniscectomy was the main risk factor negatively associated with OA changes.	N/D
Serna et al.,, 2025 [8]	Retrospective, propensity-matched cohort; Level III evidence.	ACLR alone (CPT 29888) ACLR + LEAP (CPT 29888 + 27427)	5—yr cumulative revision; 2—yr cumulative revision; differences in 5—yr in meniscus surgery, lysis of adhesions, manipulation, or 90—day ED visits	Patients (*n*) = 1022 per group; Mean age 24.2 y (ACLR + LEAP) vs. 30.0 y (ACLR); Female 46.5%; Overweight/obesity 20.1% vs. 24.9%;	5—yr revision: ACLR + LEAP 2.6% vs. ACLR 4.9%; 2—yr revision: ACLR + LEAP 0.7% vs. ACLR 3.2%	LEAP utilization rose >20% year-on-year after 2017 (except 2020); Only one TKA conversion in ACLR + LEAP group over 5 yrs; No increase in other secondary complications; Coding ensured ipsilateral procedures only.	N/D
Hopper et al., 2022 [14]	Retrospective cohort study; Level III evidence.	Isolated ACLR (primary hamstring or patellar tendon autograft); ACLR + LEAP (lateral extra-articular procedure)	Graft failure requiring revision; Contralateral ACL rupture; Ipsilateral secondary surgeries	Patients (*n*) = 342 (166ACLR + 176 ACLR + ALLR professional athletes; mean follow-up 100.2 ± 51.9 months (range 24–215); age subgroup ≤21 vs. >21 years; graft types: hamstring or patellar tendon.	ACLR:15.5%; ACLR + LEAP: 6.0% (*p* = 0.0105); HR for isolated ACLR 2.678 (95% CI 1.173–4.837; *p* = 0.0164)	Athletes ≤21 years at >2-fold risk of failure (HR 2.381; 95% CI 1.313–5.463; *p* = 0.0068); Sex, sport, graft type aren’t significant risk factors; Contralateral ACL rupture: 13.2%; Ipsilateral secondary surgeries: 18.1%	Y
Guy et al., 2022 [15]	Retrospective cohort study; Level of evidence III	Isolated ACLR ACLR + LEAP (modified Lemaire or anterolateral ligament reconstruction)	Graft rupture	*n* = 81 (50 ACLR + 31 ACLR + LEAP) elite alpine skiers; min follow-up 2 y;	ACLR: 34.0%; ACLR + LEAP 6.5% (*p* = 0.0412)	Age (HR 1.114; *p* = 0.1157), sex (HR 1.573; *p* = 0.3743) and graft type (HR 1.417; *p* = 0.5394) were not associated with a higher risk of graft rupture.	N/D
Mahmoud et al., 2022 [9]	Retrospective matched cohort study; Level III evidence;	ACLR + LEAP (iliotibial band tenodesis via modified Lemaire technique through Kaplan’s fibers); Isolated ACLR (standard semitendinosus–gracilis autograft)	PROMs (Lysholm, IKDC, OKS, Tegner), Graft Rupture Rates	Patients (*n*) = 144 (72 per group); mean age 25 ± 8.5 y; 76% male; median follow-up 10 y (IQR 6.7)	ACLR-LEAP: 5%; ACLR: 11%	PROMs (Lysholm, IKDC, OKS, Tegner) improved significantly post-op in both groups with no significant between-group differences (e.g., Lysholm Δ *p* = 0.82, IKDC Δ *p* = 0.07); Few complications: ACLR-LEAP 3 meniscus re-arthroscopies; ACLR 1 DVT, 1 superficial infection, 4 meniscus re-arthroscopies; PROMs exceeded MCID in majority of patients	N/D
Porter et al., 2020 [5]	Prospective randomized controlled trial; Level II evidence; skeletally mature patients with residual pivot shift post-ACLR.	Group A: ACLR alone (no further surgery) Group B: ACLR + Modified Iliotibial Band Tenodesis (MITBT) added intraoperatively	IKDC score; KOOS Sport/Rec; KOOS QoL; Lysholm; Tegner: median; Recurrent ACL ruptures; Meniscal tears; Contralateral ACL ruptures	Patients (*n*) = 55 randomized (27 A, 28 B); female:male A 15:12, B 17:11; mean age 22.3 ± 3.7 y (A) vs. 21.8 ± 4.1 y (B); pivoting sports; no meniscal repair.	Recurrent ACL ruptures: 14.8% (A) vs. 0% (B); *p* < 0.001	IKDC score: 90.9 ± 10.7 (A) vs. 94.2 ± 11.2 (B); *p* = 0.21; KOOS Sport/Rec: 91.5 ± 6.4 vs. 95.3 ± 4.4; *p* = 0.02; KOOS QoL: 92.0 ± 4.8 vs. 95.1 ± 4.3; *p* = 0.14; Lysholm: 92.5 ± 4.8 vs. 96.8 ± 8.0; *p* = 0.004; Tegner: median 7 [6,7,8] vs. 8 [7,8,9]; *p* = 0.03; Recurrent ACL ruptures: 14.8% vs. 0%; *p* < 0.001; Meniscal tears: 14.8% vs. 3.6%; *p* = 0.14; Contralateral ACL ruptures: 3.7% vs. 3.6%; *p* = 0.99; MITBT reduced residual pivot shift: Improved KOOS Sport/Rec, Lysholm, Tegner scores; No difference in contralateral ruptures	Y
Rowan et al., 2019 [10]	Retrospective cohort; propensity-matched case–control; Level III evidence.	ACL reconstruction alone (single-bundle doubled hamstring graft) ACL reconstruction + LEAT (modified iliotibial band tenodesis) based on high-grade pivot shift or ≥2 minor criteria	Post-op Lysholm score; Tegner activity index; Time to return to sport; ACL re-injury (ipsilateral)	Patients (*n*) = 171 (125 ACLR alone vs. 46 ACLR + LEAT (matched)) Median age 29 vs. 27 years; 54% vs. 59% male; Sports: football, skiing, rugby, hockey, basketball, others; Elite athletes	ACLR: 5%; ACLR + LEAT: 0%	Post-op Lysholm score: median 90 vs. 98; *p* = 0.005; Tegner activity index: mean 7.54 vs. 8.04; *p* = 0.003; Time to return to sport: median 8 vs. 6 months; *p* < 0.001	N/D

## 5. Discussion

Our systematic review and meta-analysis suggest that adding lateral extra-articular procedures (LEAP) to anterior cruciate ligament reconstruction (ACLR) may be beneficial in reducing the risk of graft failure, particularly in high-risk populations. Across the included studies, encompassing 7336 patients, the pooled analysis showed that ACLR alone was associated with nearly three times the risk of graft rupture compared to ACLR combined with LEAP, with a risk ratio of 2.72 (95% CI: 2.23–3.32; *p* < 0.00001) and negligible heterogeneity (I^2^ = 0%) [2,3,4,5,8,9]. Individual studies consistently demonstrated lower graft failure rates with LEAP augmentation. For instance, Monaco et al. [6] Anterior Cruciate Ligament Reconstruction with Lateral Extra-Articular Tenodesis Reduces Knee Rotation Laxity and Graft Failure Rate: A Systematic Review and Meta-Analysis reported 0% graft failure in adolescents undergoing ACLR + LEAP compared to 15% in isolated ACLR [2], while Borque et al. [11] observed 3.4% versus 9.5% in elite athletes. Similarly, Getgood et al. [4] found a reduction from 11.3% to 3.7% in young, high-risk patients, and Viglietta et al. [7] demonstrated a long-term decrease in graft rupture from 10.4% to 1.2%. Although a few studies such as Parmar et al. [13] reported slightly higher failure rates in LEAP cohorts, these differences were not statistically significant.

Beyond graft failure, LEAP may improve rotational stability and functional outcomes. Studies reported lower pivot-shift grades and reduced anteroposterior laxity in combined ACLR + LEAP groups, with improved Tegner and Lysholm scores in several cohorts [2,3,5,15,20]. Return-to-sport rates were comparable or superior with LEAP, with Rowan et al. [10] documenting faster return to pivoting sports and higher functional scores. Adverse event profiles were similar between groups, with no significant increase in postoperative stiffness, infection, or contralateral ACL injuries [3,15,16]. Long-term concerns regarding lateral compartment osteoarthritis were inconsistently reported; while Castoldi et al. [16] suggested a trend toward higher osteoarthritis risk after LEAP, other long-term studies found no such association and even reported lower osteoarthritis grades in LEAP groups [7,13,14]. These findings support the notion that the reduction in graft failure may contribute to long-term joint preservation and potentially reduce the risk of post-traumatic osteoarthritis, although further longitudinal studies are needed.

Recent intra-operative findings further underscore the severe long-term consequences of persistent ACL deficiency. Passaretti et al. [21] performed an intra-operative analysis in patients undergoing total knee arthroplasty and found significant correlations between ACL lesions and advanced gonarthrosis, suggesting that unaddressed rotational or translational knee instability may accelerate joint degeneration and lead to end-stage osteoarthritis requiring joint replacement. This evidence reinforces the idea that reducing graft failure and residual instability via adjunct procedures such as LEAP may not only prevent revision surgery, but also contribute to long-term joint preservation.

Potential modifying factors should be considered when evaluating LEAP. Age, participation in pivoting sports, generalized ligamentous laxity, and graft choice may influence outcomes. Adolescents and elite athletes appear to derive the greatest benefit from LEAP [3,4,6,7,11,14], and evidence suggests protective effects across hamstring, quadriceps, and bone-patellar tendon-bone grafts [4,6,12,20].

While the primary focus is on graft protection, clinicians should also consider surgical complexity, operative time, and rehabilitation implications. LEAP is an additional procedure that may increase surgical duration and requires careful technique to avoid lateral overconstraint. Rehabilitation protocols are largely similar to standard ACLR, but close monitoring for lateral knee pain or stiffness is recommended [3,7]. Cost-effectiveness analyses remain limited; however, the potential to reduce revision surgeries may provide long-term economic benefits.

In conclusion, the current evidence suggests that LEAP may be beneficial in reducing graft failure and improving rotational stability in selected high-risk patients without a significant increase in complications. These findings support a selective application of LEAP, guided by patient-specific risk factors, intraoperative assessment, and consideration of modifying factors such as age, sport, and graft type [2,4,6,7,11,21]. Future studies should further investigate long-term joint preservation, cost-effectiveness, and optimal patient selection criteria.

## Figures and Tables

**Table 1:** Graft Rupture.

Study	ACLR	ACLR + LEAP
Monaco et al., 2022 [6]	15%	0%
Borque et al., 2022 [11]	9.5%	3.4%
Heard et al., 2023 [3]	11%	4%
Mao et al., 2021 [17]	12%	3.93%
El-Azab et al., 2023 [19]	6.3%	2.1%
Feng et al., 2022 [18]	11.5%	3.6%
Castoldi et al., 2020 [16]	29%	13%
Getgood et al., 2020 [4]	11.3%	3.7%
Brinkman et al., 2025 [12]	HA 17.9%, QA 1.8%	4.3%
Parmar et al., 2025 [13]	3%	4.7%
Lucidi et al., 2025 [20]	37%	19%
Viglietta et al., 2022 [7]	10.4%	1.2%
Serna et al., 2025 [8]	4.9%	2.6%
Hopper et al., 2022 [14]	15.5%	6%
Guy et al., 2022 [15]	34.0%	6.5%
Mahmoud et al., 2022 [9]	11%	5%
Porter et al., 2020 [5]	14.8%	0%
Rowan et al., 2019 [10]	5%	0%

## Data Availability

Data supporting the findings of this study are available from the corresponding author upon reasonable request.

## Supplementary Materials

The following supporting information can be downloaded at: https://www.mdpi.com/article/10.3390/jcm14238499/s1, PRISMA Checklist.
